# Seroprevalence of SARS-CoV-2 Neutralizing Antibodies among Blood Donors in Ho Chi Minh City, Vietnam, August–November 2020

**DOI:** 10.4269/ajtmh.21-0259

**Published:** 2022-01-26

**Authors:** Hanh Hong Ho Nguyen, Quan Hoang Nguyen, Dung Thi Thuy Truong, Manh Huy Dao, Tu Ngoc Le, Hieu Trung Nguyen, Anh Hoang Nguyen, Thinh Viet Nguyen, Dao Thi Nhu Hoang, Loan Kim Hoang, Tham Thi Tran, Thang Minh Cao, Quang Chan Luong, Lan Trong Phan, Loan Thi Kim Huynh, Thuong Vu Nguyen, Quang Duy Pham

**Affiliations:** ^1^ Training Center, Pasteur Institute of Ho Chi Minh City, Ho Chi Minh City, Vietnam;; ^2^ School of Medicine, Vietnam National University, Ho Chi Minh City, Vietnam;; ^3^ Viet-Anh Department, Hospital for Tropical Diseases, Ho Chi Minh City, Vietnam;; ^4^ Microbiology and Immunology Department, Pasteur Institute of Ho Chi Minh City, Ho Chi Minh City, Vietnam;; ^5^ Department for Disease Control and Prevention, Pasteur Institute of Ho Chi Minh City, Ho Chi Minh City, Vietnam;; ^6^ Blood Donation Centre of Ho Chi Minh City, Ho Chi Minh City, Vietnam;; ^7^ Pasteur Institute of Ho Chi Minh City, Ho Chi Minh City, Vietnam;; ^8^ Planning Division, Pasteur Institute of Ho Chi Minh City, Ho Chi Minh City, Vietnam

## Abstract

Relatively little is known about the seroprevalence of severe acute respiratory syndrome coronavirus 2 (SARS-CoV-2) IgG antibodies and COVID-19-related behaviors in the general population in Vietnam, where the first case of COVID-19 was detected on January 22, 2020. We surveyed a group of 885 blood donors at community blood donation sessions in Ho Chi Minh City from August 27 to November 7, 2020. Blood was collected to test for SARS-CoV-2 IgG antibodies using the plaque reduction neutralization test. We adjusted the seroprevalence by weight for ages 18 to 59 years old obtained from the 2019 population census. The weighted seroprevalence estimate for SARS-CoV-2 neutralizing IgG antibodies was 0.20% (95% CI, 0.05–0.81). Reports of usually or always using a mask in public places were observed at high levels of 28.6% and 67.5%, respectively. The percentages of usually or always washing hands with soap or disinfecting with hand sanitizer after touching items in public places were 48.0% and 37.6%, respectively. Although our findings suggest undocumented exposure to the virus, the seroprevalence of SARS-CoV-2 IgG antibodies among blood donors was low in this city.

## INTRODUCTION

Vietnam was widely recognized as a country with successful stories in responding to COVID-19.^1^ In this country, the first case of COVID-19 was detected on January 22, 2020,^2^ and the first community transmission was confirmed on March 18, 2020.^3^ At the time of writing in early March 2021, there have been just 2,472 cumulative cases and 35 reported deaths, representing 2.5 cases and 0.04 deaths per 100,000 people in Vietnam.^4^ These case-notified data, however, are unable to reflect the true extent of transmission of severe acute respiratory syndrome coronavirus 2 (SARS-CoV-2) in the country. Despite the few serological studies in Vietnam,^5^^,^^6^ no representatively obtained data on the SARS-CoV-2 seroprevalence among the general population exist.

The objective of our study was to determine the seroprevalence rate of anti-SARS-CoV-2 antibodies among blood donors recruited from community blood donation sessions in Ho Chi Minh City, Vietnam.

## MATERIALS AND METHODS

### Study design and participants.

This cross-sectional study with the target sample size of 900 voluntary blood donors was conducted approximately 5 months after the peak of the first epidemic wave in Ho Chi Minh City in March 2020. To obtain 80% power at a two-sided 5% significance level for estimating a seroprevalence of 1.0% for an assumed half-width 95% confidence limit of 1.0% and a design effect of two, the required sample size was 761, and it was rounded to 900 to allow for declined interviews and damage or loss of blood specimens.

**Figure 1.**
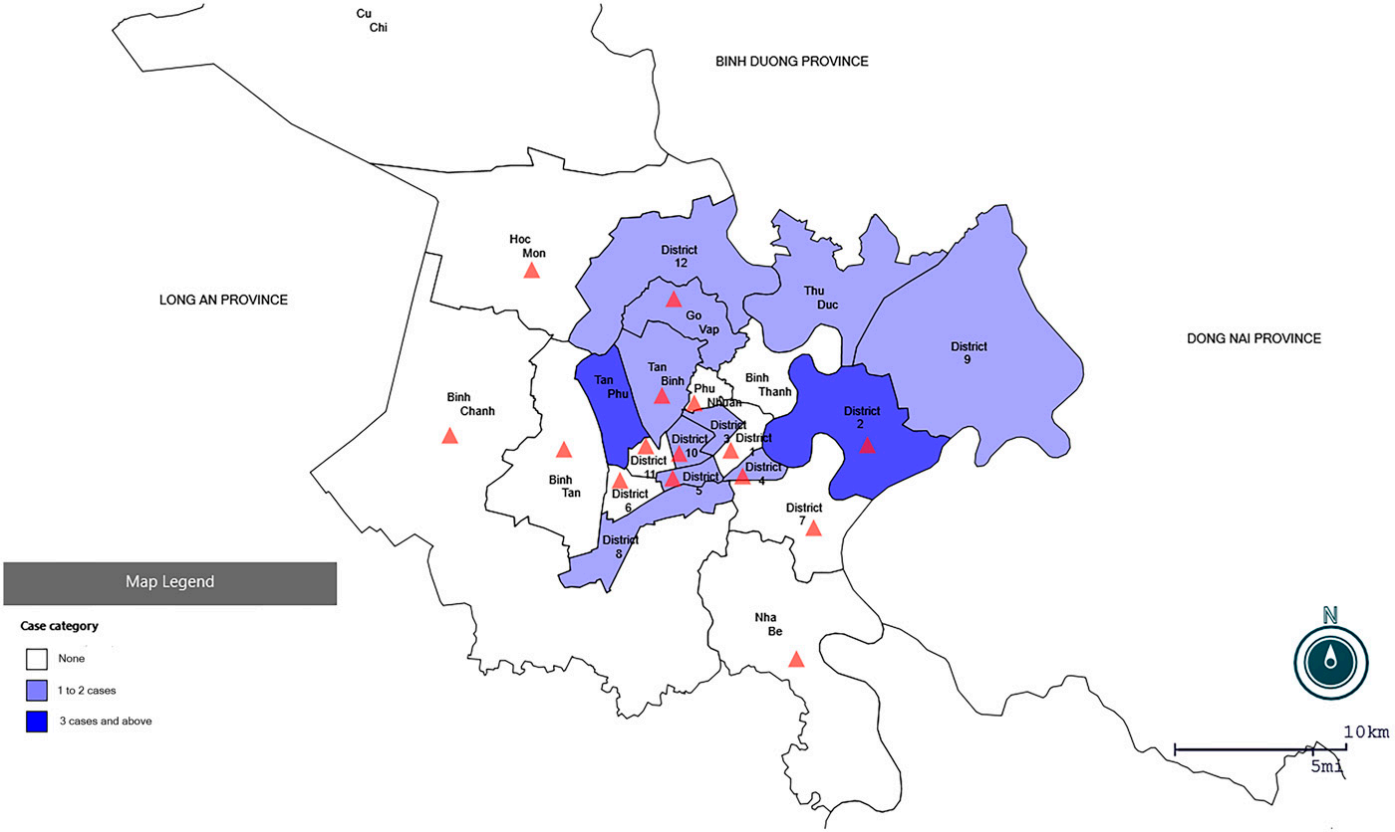
Map showing the location of districts selected for the severe acute respiratory syndrome coronavirus 2 serosurvey, Ho Chi Minh City, Vietnam, August through November 2020. The triangles represent the selected districts. This figure appears in color at www.ajtmh.org.

We developed a sampling frame for this survey in Ho Chi Minh City using the WHO generic protocol for COVID-19 seroprevalence.^7^ Based on 28 locally acquired infections identified in this city prior to recruitment initiation, we categorized the city’s 24 administrative districts into three groups: districts with three or more cases (two districts), districts with two cases (four districts), and districts with zero to one case (18 districts). These three groups accounted for 50%, 30%, and 20% of the total cases, respectively. To cover areas with at least 60% of the 24 districts with both the high and low numbers of reported cases, we aimed to select 15 districts, which were chosen randomly from three district groups (www.random.org/). This selection included one of two districts with three or more locally acquired cases, three of four districts with two cases, and 11 of 18 districts with zero to one case (Figure 1). Each district from this study targeted enrolment of 60 blood donors stratified by age group (18–19, 20–29, 30–39, 40–49, and 50–59 years) and gender based on the 2019 population census data of Ho Chi Minh City.

Eligibility criteria for this study were voluntarily donating blood at blood donation sessions in the community, age 18 to 59 years, living in Ho Chi Minh City for 3 months or more, and willingness to provide informed consent. In these community blood donation sessions, we were able to recruit donors from varied and diverse backgrounds and income, including students, housewives, blue- and white-collar workers, shippers, drivers, police officers, teachers, retailers, waiters/waitresses, health-care workers, and so on. At each blood donation session, participants were recruited consecutively until the targeted sample size of 60 individuals was reached. We excluded from the analysis individuals who refused to be interviewed for collecting study information.

### Data collection.

A 7.5-mL blood specimen was collected from donated blood bags for those who were eligible to donate blood, or was collected from a vein for those who were deferred from blood donation. Participants then completed a behavioral interview [including demographic information, medical conditions, travel history, COVID-19 knowledge and behaviors, signs and symptoms they experienced in the previous 6 months, and self-perception of risk of SARS-CoV-2 infection (Supplemental Appendix S1)].

Blood specimens were tested for the presence of the neutralizing IgG antibodies to SARS-CoV-2 using plaque reduction neutralization tests (PRNTs) (Supplemental Appendix S1). Using the log_2_-dilution series, we initiated the PRNTs with a 1:10 serum dilution. Serum dilutions causing a 50% reduction in the number of plaque counts (PRNT_50_) were considered as titers. We also selected and tested 46 pre-pandemic sera of blood donors collected in 2017 to establish the specificity of the PRNT. Testing results were returned to the participants via phone 3 months after enrollment.

### Statistical analysis.

Data obtained were subject to descriptive analysis. Unlike a relatively equal gender distribution, age distribution differed between the blood donor study sample and the population by age group (18–19, 20–29, 30–39, 40–49, and 50–59 years) in Ho Chi Minh City (Supplemental Table S1). As a result, we weighted the overall seroprevalence estimates of the neutralizing IgG antibodies to SARS-CoV-2 with 95% CIs for ages 18 to 59 years by using the census midyear 2019 population estimate for 18- to 59-year-old people for Ho Chi Minh City (Supplemental Appendix S1).

### Ethical consideration.

Our study was approved by the institutional review board of the Pasteur Institute of Ho Chi Minh City (reference no. 05/GCN-PAS). All participants provided written informed consent.

## RESULTS

From August 27 to November 7, 2020, 928 blood donors were screened for the study. Of these donors, 906 met inclusion criteria for the study, 895 (98.8%) blood specimens were collected, and 887 (97.9%) completed the interviews. In total, 885 of 906 participants (97.6%) who provided both behavioral and serological data were included in our analysis. Among the 885 blood donors, four (0.5%) were deferred donors for staying up late the night before donation, being underweight, and having a short donation interval (< 12 weeks).

For the 885 blood donors analyzed, the median age was 35 (interquartile range [IQR], 25–46) years, and 432 individuals (48.8%) were men. In all, 80.8% (705 of 872) completed high school education or more, and 74.0% (654 of 884) were employed or self-employed. The median monthly income of the employed or self-employed participants was US$351 (range, US$20–4,392). In this sample, the median number of household members was four people (IQR, 3–5). Fifty-six of the 884 blood donors (6.3%) reported having had underlying medical conditions (Table 1).

**Table 1:** Samples characteristics, travel history, and risk behaviors related to COVID-19 among blood donors in Ho Chi Minh City, Vietnam, August 27 to November 7, 2020 (*N* = 885)

Characteristic	Value
Demographic	
Age, y; median (IQR); (*n* = 885)	35 (25–46)
Male gender, *n *(%); (*n* = 885)	432 (48.8)
Highest education attained, *n *(%); (*n* = 872)	
Primary	45 (5.2)
Secondary	122 (14.0)
High school	267 (30.6)
College or university	438 (50.2)
Marital status, *n *(%); (*n* = 882)	
Never married	370 (42.0)
Married	502 (56.9)
Divorced	10 (1.1)
Current labor force status, *n *(%); (*n* = 884)	
Employed or self-employed	654 (74.0)
Unemployed	230 (26.0)
Average monthly income,* US$; median (range) (*n* = 618)	351 (20–4,392)
No. of household members, median (IQR); (*n* = 883)	4 (3–5)
No. of people sharing a bedroom, median (IQR); (*n* = 881)	2 (1–3)
Have underlying medical conditions,† *n *(%); (*n* = 884)	56 (6.3)
Travel history, *n *(%)	
Traveled to other provinces in the previous 6 months (*n* = 883)	198 (22.4)
Traveled oversea in the previous 6 months (*n* = 883)	4 (0.5)
Traveled to the Da Nang outbreak region (*n* = 883)‡	18 (2.0)
Behaviors related to COVID-19 infection	
Visited a health facility in the previous 6 months, *n *(%) (*n* = 883)	313 (35.4)
Attended a gathering of ≥ 10 people since the start of the outbreak, *n *(%) (*n* = 879)	411 (46.8)
Attended a gathering of ≥ 10 people in the previous 30 days, *n *(%) (*n* = 878)	380 (43.3)
Used a mask in public places (*n* = 880) , *n *(%)	
Always	593 (67.5)
Usually	252 (28.6)
Sometimes	32 (3.6)
Rarely	2 (0.2)
Never	1 (0.1)
Used a mask in the 5 days prior to enrollment, *n *(%)	
Day 1 (*n* = 883)	708 (80.2)
Day 2 (*n* = 883)	718 (81.3)
Day 3 (*n* = 883)	715 (81.0)
Day 4 (*n* = 883)	721 (81.7)
Day 5 (*n* = 883)	842 (95.4)
Washed hands with soap and water or disinfected with hand sanitizer after touching items in public places, *n *(%); (*n* = 882)	
Always	332 (37.6)
Usually	423 (48.0)
Sometimes	104 (11.8)
Rarely	13 (1.5)
Never	10 (1.1)
Average no. of people with whom participants had any contact at a physical distance of less than 2 m (6 feet) on a daily basis, median (IQR); (*n* = 879)	8 (4–15)
Used public transport to work, *n *(%); (*n* = 882)	16 (1.8)
Had a household co-inhabitant with fever or an upper respiratory illness in the previous 6 months, *n *(%); (*n* = 885)	105 (11.9)
Self-perceived to be high risk of acquiring COVID-19, *n *(%); (*n* = 861)	57 (6.6)

IQR = interquartile range.

* Monthly income was obtained from participants who were employed or self-employed only.

† Participants were asked whether they had any underlying medical conditions associated with COVID-19, including chronic pulmonary diseases, cardiovascular diseases, high blood pressure, diabetes, and other chronic disorders.

‡ Approximately 1 month before study recruitment initiation, Vietnam faced its first substantial community transmission of severe acute respiratory syndrome coronavirus 2 occurring in Da Nang during July and August 2020.

A small proportion of blood donors had traveled overseas (0.5%, 4 of 883) or had returned from Da Nang, where the first substantial community transmission of COVID-19 had occurred during July and August 2020 (2.0%, 18 of 883), about 1 month before the start of our study. In addition, more than one third (35.4%, 313 of 883) had visited a health facility in the previous 6 months.

Of the 880 blood donors who reported mask use frequency, 593 (67.5%) reported always wearing a mask and 252 (28.6%) reported usually wearing a mask. During the 5 days before being interviewed, the use of facemasks dropped from 95.4% to 80.2%. In addition to mask use, reports of usually or always washing hands with soap or disinfecting with hand sanitizer after touching items in public places were 48.0% and 37.6% of participants, respectively. The median daily number of people with whom participants had any contact at a physical distance of less than 2 m (6 feet) was eight people (IQR, 4–15). Of the 885 blood donors, 105 (11.9%) reported their family members had fever or an acute upper respiratory tract illness in the previous 6 months. A self-perception of being at high risk of SARS-CoV-2 infection was 6.6% (57 of 861) (Table 1).

SARS-CoV-2 neutralizing IgG antibodies to SARS-CoV-2 were identified in two blood donors (0.23%; 95% CI, 0.06–0.82), whereas none of 46 pre-pandemic stored sera tested positive for anti-SARS-CoV-2 antibodies using PRNT_50_ (100% specificity; 95% CI, 92.3–100%). After being adjusted for ages 18 to 59 years of the midyear population census estimate for the 18- to 59-year-old population for Ho Chi Minh City, the weighted seroprevalence rate was 0.20% (95% CI, 0.05–0.81) (Table 2).

**Table 2:** Unweighted and weighted seroprevalence rates of the severe acute respiratory syndrome coronavirus 2 neutralizing IgG antibodies among blood donors by age group in Ho Chi Minh City, Vietnam, August 27 to November 7, 2020

Age group, y	No. of participants	Confirmed cases with neutralizing IgG antibodies, *n*	Unweighted seroprevalence rate, % (95% CI)	Weighted seroprevalence rate, (95% CI)
18–19	79	0	0.00 (0.00–4.64)	0.00 (n/a)
20–29	245	1	0.41 (0.08–2.28)	0.41 (0.06–2.9)
30–39	227	1	0.44 (0.08–2.45)	0.44 (0.06–3.1)
40–49	191	0	0.00 (0.00–1.97)	0.00 (n/a)
50–59	143	0	0.00 (0.00–2.62)	0.00 (n/a)
Total	885	2	0.23 (0.06–0.82)	0.20 (0.05–0.81)

n/a = not applicable.

One participant had a PRNT_50_ titer of 1:80 and another had a PRNT_50_ titer of 1:10. There was no epidemiological link between these cases. Both participants reported they had not traveled to known outbreak areas, and they had not had any known contact with any person returning from such an area and any suspected COVID-19 cases. They further reported that about 1 to 2 months before blood donation, they had experienced an episode of suspected illness characterized by cough and sore throat in one case, and fever and fatigue in the other.

## DISCUSSION

The low seroprevalence rate of anti-SARS-CoV-2 antibodies observed in Ho Chi Minh City is comparable to the estimates reported among communities in outbreak areas in Hanoi, Quang Nam, and Da Nang (0.20%).^6^ This low seroprevalence was consistent with the low positivity rate for respiratory specimens by polymerase chain reaction, which was estimated to be 0.016% (13 of 80,395) for the survey period in this city. Low seroprevalence rates were described previously in other countries pursuing a zero to low COVID-19 transmission policy, such as 0.05% among hospital patients in Taiwan^8^ and 0.07% among outpatients in South Korea.^9^

Early in the pandemic, Vietnam closed borders with China, suspended international flights, conducted aggressive testing, isolated cases, conducted contact tracing, enacted mass quarantine of close contacts, and rapidly imposed a national lockdown.^10^^,^^11^ Since mid-March 2020, this country mandated wearing masks in public places and promoted handwashing practices. Mask use was reported at levels of 90% in early August 2020 following reports of the outbreak in Da Nang.^12^ The early and aggressive response, adaptive preventive behaviors in the community, and the warm and wet climate of local settings could have helped to limit the wide spread of SARS-CoV-2 in Ho Chi Minh City.^11^^,^^13^ The low seroprevalence rate suggests that Vietnam’s strategies during the first wave of the epidemic was effective compared with many countries in other parts of the world.

With 0.20% of the blood donor population having the neutralizing IgG antibodies to SARS-CoV-2, the scale of the COVID-19 epidemic in Ho Chi Minh City, a city of approximately 9 million people, is likely to be greater than the case notification we observed of 166 reported cases by the end of 2020 (Supplemental Figure S1). Our interview with the two undocumented blood donors having antibodies against SARS-CoV-2 revealed they had experienced subclinical or mild symptoms 1 to 2 months prior to blood donation. Taken together, there has been a potential risk for community transmission of SARS-CoV-2 in Ho Chi Minh City. In May 2021, a church was the center of a COVID-19 outbreak caused by the delta variant in Ho Chi Minh City. As a result, the number of COVID-19 cases and deaths spiked in this city. As of September 29, 2021, this outbreak has resulted in more than 376,600 cases and 14,600 COVID-19-related deaths.^14^ Before this outbreak, the city’s testing policy focused solely on testing international travelers, close contacts of a confirmed COVID-19 case, and suspected cases living in active outbreak areas. Therefore, there is a need for an extension of symptomatic testing in Ho Chi Minh City. Symptomatic testing along with efficient contact tracing has shown the potential to reduce the risk of community transmission of SARS-CoV-2.^15^^,^^16^

This study has several major limitations. First, we only recruited adult blood donors in one city, so the findings cannot be generalized widely. Second, the healthier status of the blood donor population than the general population may lead to underestimating the true extent of a seroprevalence rate of anti-SARS-CoV-2 antibodies in the community.^17^ Third, an inability to recruit all occupations with various risks of COVID-19 and assess participants’ access to personal protect equipment in our sample may result in potential biases in estimating the seroprevalence reported. Fourth, because neutralizing antibodies peak 31 to 35 days after symptom onset and wane overtime,^18^ the use of the PRNT as a measure of seropositivity is less likely to detect declining antibody levels because it is more specific but less sensitive. Although PRNT is a gold standard for detecting neutralizing antibody response, this time-consuming technique requires a certain set of skills from staff performing experiments with the live virus in a biosafety level 3 laboratory.^19^ Future serological studies using binding assays rather than PRNTs are thus highly warranted.^20^

In conclusion, our finding highlights the importance of serosurveys of blood donors in monitoring the transmission pattern and epidemiological trends of SARS-CoV-2 transmission. Evidence of an undocumented exposure to SARS-CoV-2 suggests additional enhancement of the active and passive surveillance systems, and a continued commitment of rapid response in case of a reoccurrence of COVID-19 outbreaks in this city.

## Supplemental Material


Supplemental materials
