# Supplementary material for: Seroprevalence of SARS-CoV-2 Neutralizing Antibodies among Blood Donors in Ho Chi Minh City, Vietnam, August–November 2020
Source: Am J Trop Med Hyg. 2022 Jan 26;106(3):891–5. doi: 10.4269/ajtmh.21-0259 (PMC8922498; doi:10.4269/ajtmh.21-0259)
Supplement: Supplementary file 1 [file tpmd210259.SD1.pdf]

Supplemental Appendix.

1. Specimens handling and testing

A 7.5 ml of blood was collected from blood donors at blood donation sessions in the community in Ho Chi Minh City. The whole-blood specimens were transported within 4 hours after collection to a laboratory at the Pasteur Institute in Ho Chi Minh City for extracting and aliquoting serum into five tubes (0.5 mL each). Sera were then stored at -70°C and were inactivated at 56°C for 30 minutes prior to performing plaque reduction neutralization testing (PRNT).

Plaque reduction neutralization testing

The PRNT assay was carried out to determine the neutralizing antibodies against SARS-CoV-2 as per the methods of Thomas et al. (*1*) and Nguyen et al (*2*). In brief, for this assay, we cultured Vero E6 cells in a 12-well plate (Corning) using strains previously isolated from a family cluster (*3*) (GISAID accession numbers MT192772 and MT192773) and the log_2-dilution series approach commencing 1/10. At each dilution, 0.15 mL of inactivated serum was mixed with 0.15 mL virus, each microliter of which had 2500 plaque-forming units. The mixed products were incubated at 37°C for 1 hour. After the incubation period, 50 µL of the mixture was added into the monolayers of Vero E6 cells and additionally incubated at 37°C in 5% CO_2 for 1 hour. 1-2 mL overlay medium (DMEM with 1% methylcellulose (Wako Pure Chemical Industry), in 2% FBS) was prepared and added into each well of a plate. The plate was subsequently incubated at 37°C in 5% CO_2 for 5–6 days until plaques were established. Cells were fixed by using 10% formaldehyde buffer for 2 hours at RT and stained with 0.45% methylene blue

tetrahydrate (Wako). We manually counted the plaques. Serum dilutions causing a 50% reduction in the number of plaque counts ($PRNT_{50}$) were considered as a titer. We considered the presence of neutralizing antibodies if the $PRNT_{50}$ titer was ≥10.

2. Weighting methods for seroprevalence

Exploratory data analysis found that sex was equally distributed between the blood donor study sample and the population. Age distribution differed between the blood donor study sample and the population by age groups (18-19, 20-29, 30-39, 40-49, and 50-59 years) in Ho Chi Minh City (Table S1). Therefore, we estimated the seroroprevalence of IgG neutralizing antibodies for age groups using *svy* procedures (pweight) in Stata 14.0. For each age group, a weight was calculated by dividing the population proportion by the sample proportion (Table S1).

Table S1. Demographics of the study sample and the population in Ho Chi Minh City obtained from the 2019 Vietnam Population and Housing Census.

**Characteristic**	**Blood donor sample**		**HCMC's population***		**Weights†**
	**N**	**%**	**N**	**%**	
**Age group (years)**					
18-19	79	8.9%	309,446	5.1%	0.568
20-29	245	27.7%	1,790,659	29.3%	1.060
30-39	227	25.6%	1,745,832	28.6%	1.115
40-49	191	21.6%	1,309,359	21.5%	0.994
50-59	143	16.2%	946,859	15.5%	0.960
**Sex**					
Males	432	48.8	2,968,988	48.7	-
Females	453	51.2	3,133,167	51.3	-

*Data were obtained from the 2019 Vietnam Population and Housing Census (4). We calculated the 18-19 year old population in HCMC by multiplying the overall 15-19 year old population Ho

Chi Minh City (HCMC) by the proportion of the 18-19 year old population in the whole eastern region, including HCMC and other provinces.

†Weight was calculated by dividing the population proportion by the sample proportion.

3. Epidemic trends of COVID-19 in Ho Cminh City

Figure S1. Epidemic curve of COVID-19 cases in Ho Chi Minh City, Vietnam, 23 January 2021 – 19 April 2021. Cases are indicated by diagnosis data.

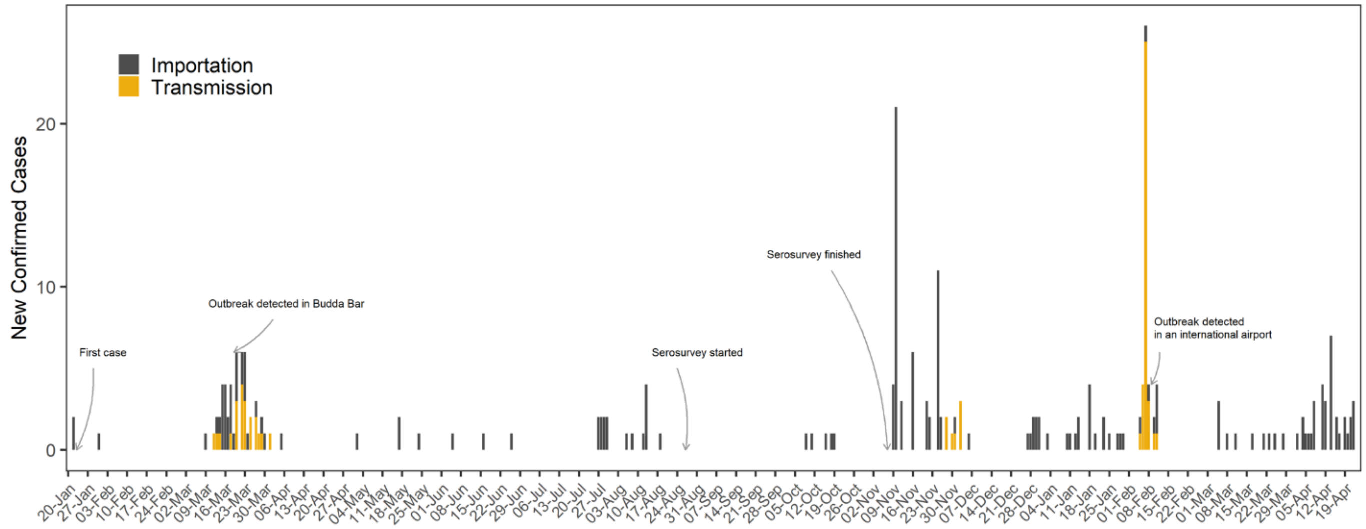

4. Study Questionnaire

Interview date: ____/____/_______

Interviewer: _________________ **Signature:** ________

Collection facility: ____________ **Object Code**: [Paste code here]_________________________

1. DEMOGRAPHIC CHARACTERISTICS:

**Date of birth**<br>***If do not remember correctly, age is acceptable:***	____/____/____ (If not remember correctly, fill year only)<br>___________ age
**Gender**	☐ Male ☐ Female
**Marital status**	☐ Single ☐ Married ☐ Other: _____________
**Education**	Number of educated years: _____________ ☐ Illiteracy
**Residence**	Address: ___________________________<br>Wards: ___________________________<br>District: ___________________________
**Ethnic groups**	☐ Kinh ☐ Chinese-born ☐ Other (specify): ____________

2. MEDICAL HISTORY (from childhood to present):

**Do you smoke cigarette?**<br>-If yes, specify quantity:	☐ Currently smoking<br>*Smoking duration, until dated: _____ ☐ day / ☐ week / ☐ month / ☐ year*<br>*Average number of cigarettes smoked/day in the last 30 days: ______*<br>☐ Quitted<br>*Years smoked: ______ ☐ month / ☐ year*<br>☐ Never smoke
**Do you drink alcohol/beer?**<br>-If yes, specify quantity:	☐ Drank recently<br>*Number of times in the last 30 days: ______ times*<br>*How much each time (average): ___________ ☐ Alcohol / ☐ beer*<br>*(Specify units, bottles or cans with beer; liters with alcohol....)*<br>☐ Used to drink quitted<br>☐ Never drank alcohol or beer
**Do you have any chronic diseases?**	☐ Yes<br>☐ No
**History of chronic pulmonary disease**<br>-If yes, enter the number of months and years of illness:	☐ Yes, specify ________<br>Duration: ____ years ____ months________<br>☐ No ☐ Unknown
**History of cardiovascular disease**<br>-If yes, enter the number of months and years of illness:	☐ Yes, specify ________<br>Duration: ____ years ____ months__<br>☐ No ☐ Unknown
**History of high blood pressure**<br>-If yes, enter the number of months and years of illness:	☐ Yes, specify ________<br>Duration: ____ years ____ months__<br>☐ No ☐ Unknown
**History of diabetes**<br>-If yes, enter the number of months and years of illness:	☐ Yes, specify ________<br>Duration: ____ years ____ months__<br>☐ No ☐ Unknown
**Other illness**<br>-If yes, enter the number of months and years of illness:	☐ Yes, specify ________<br>Duration: ____ years ____ months__<br>☐ No ☐ Unknown
**Blood transfusion:**<br>-If yes, enter month and year of most recent blood transfusion	☐ Yes ☐ No ☐ Unknown<br>____/________

3. RISK EXPOSURE FACTORS:

In the past 6 months, have you **traveled <u>domestically?</u>**<br>If yes, specify the location, the date (from/to):	☐ Yes ☐ No<br><br>Destination: ________________________<br>Time: ________________________
Have you been to **Da Nang?**<br>If yes, specify the location, the date (from/to):	☐ Yes ☐ No<br>Destination: ________________________<br>Time: ________________________
In the past 6 months, **have you visited any <u>medical facility</u>?**<br>If yes, specify the reason for coming (for medical examination, visit...), specify the time spent:	☐ Yes ☐ No<br>**<u>If yes</u>**, reasons: ☐ Work ☐ Treatment ☐ Examination<br>☐ Visit patient ☐ Other: ___________<br>Name of medical facility: _______________<br>Time spent: ________ ☐ day / ☐ week / ☐ month / ☐ year<br>☐ Daily
In the past 6 months, have you **<u>traveled abroad</u>?**<br>If yes, specify the location, the date (from/to):	☐ Yes ☐ No<br>Destination: ________________________<br>Time: ________________________
Type of residence within the last 6 months:	☐ Apartment ☐ Hostel ☐ Private home ☐ Other, specify: ________
Number of members in the family<br>Number of members in the same bedroom?	Number of family members: ________ person(s)<br>Number of members in the same bedroom: _______ person(s)
Since the onset of COVID19 outbreak in February 2020 until now, how often have you been **with large crowd (>10 people)?** (Conference, wedding party, bar/pub, karaoke…)	☐ Yes ☐ No<br><br>If yes, did you wear a mask?<br>☐ Always ☐ Frequently ☐ Sometimes ☐ Rarely ☐ Never
**In the past 30 days, how often did** you go to places with **large gatherings (>10 people)?** (Conference, wedding party, bar/pub, karaoke…)	☐ Yes ☐ No<br><br>If yes, did you wear mask?<br>☐ Always ☐ Frequently ☐ Sometimes ☐ Rarely ☐ Never
Since the outbreak of COVID19 from February 2020, how often do you wear **a mask in public places?**	☐ Always ☐ Frequently ☐ Sometimes ☐ Rarely ☐ Never
What kind of mask do you usually wear?	☐ Medical mask ☐ Cloth mask<br>☐ Do not carry ☐ Other: ___________

If you do not wear a mask, please explain the reason?	☐ I often forget to wear it ☐ I feel uncomfortable wearing it<br>☐ Do not know place to buy masks ☐ The price is too high<br>☐ Other reasons: ___________
In the last 5 days, please mark the days when you wore a mask in public.	☐ Day 1 (*today)*<br>☐ Day 2<br>☐ Day 3<br>☐ Day 4<br>☐ Day 5
Since the outbreak of COVID19 from February 2020, how often did you **wash your hands with soap or use a hand sanitizer** after touching items in public places?	☐ Always<br>☐ Frequently<br>☐ Sometimes<br>☐ Rarely<br>☐ Never
On average, how many times per day do you wash your hands with soap/sanitizer?	Number of wash hands time per day: _____________
Workplace environment?	☐ Office ☐ Factory / workshop ☐ Outdoor<br>☐ At home ☐ Other: _____________
Average number of that you come in close contact (<2 meters) in on a daily basis?	Number of people: ______
What means of transportation do you use every day?	☐ Motorcycle / Bike ☐ Car ☐ Bus<br>☐ Other: __________________
In the past 6 months, what means of public transportation do you travel for long distance?<br>If yes, specify time of departure and destination:	☐ Did not travel<br>☐ Plane ☐ Train ☐ Coach ☐ Other: ______<br><br>Time: ____________ Destination: _____<br>Time: ____________ Destination: _____
Have you ever been exposed to a suspected or confirmed case of COVID-19?	☐ Having ☐ Never ☐ Unknown<br>If yes, specify the latest contact date: ___/___/___
In 6 months up until today, have you ever been quarantined?	☐ Never<br>☐ Has been ☐ Quarantined centrally ☐ Quarantined at home<br>Reason for being quarantined: _____________

In 6 months past, did you know any people infected with SARS-CoV-2?		Did this person go to a medical facility?	(Day/month/year)	Describes all the symptoms (Refer below table for more detail)
**Husband/Wife /Lover**	☐ Yes ☐ No	☐ Yes ☐ No	____________	____________
**Kids**	☐ Yes ☐ No	☐ Yes ☐ No	____________	____________
**Parents**	☐ Yes ☐ No	☐ Yes ☐ No	____________	____________
**Neighbors**	☐ Yes ☐ No	☐ Yes ☐ No	____________	____________
**Friends / Relatives**	☐ Yes ☐ No	☐ Yes ☐ No	____________	____________
**Colleagues**	☐ Yes ☐ No	☐ Yes ☐ No	____________	____________
**Other (specify): _____**	☐ Yes ☐ No	☐ Yes ☐ No	____________	____________
Ask if the people listed below had any symptoms of illness:		1. Fever<br>2. Chills<br>3. Tired<br>4. Nausea/vomiting<br>5. Dizziness<br>6. Diarrhea	7. Cough<br>8. Wheezing<br>9. Difficulty breathing<br>10. Runny nose<br>11. Sore throat	12. Muscle pain<br>13. Headache<br>14. Pain behind the eye socket<br>15. Joint pain<br>16. Chest pain<br>17. Stomachache
**In 6 months past, have anyone in your house got sick?**		**Did this person go to a medical facility?**	**(Day/month/year)**	**Specify each person's symptoms**
**Husband and wife**	☐ Yes ☐ No	☐ Yes ☐ No	____________	____________
**Kids**	☐ Yes ☐ No	☐ Yes ☐ No	____________	____________
**Parents**	☐ Yes ☐ No	☐ Yes ☐ No	____________	____________
**Grand-parents**	☐ Yes ☐ No	☐ Yes ☐ No	____________	____________
**Other (specify):______**	☐ Yes ☐ No	☐ Yes ☐ No	____________	____________

4. CLINICAL EXAMINATION:

Body weight:	______(kg)	
Height:	_____(cm)	
From January 2020 to present, do you have any of the following symptoms?	**Present**	**Past**<br>*(from 1/2020-before enrollment)*

If yes, please specify: (choose multiple)<br><br>(The interviewer read out all the symptoms to the participants)	☐ Yes ☐ No<br>☐ Unknown<br>*If yes: tick symptoms*<br>☐ Fever ☐ Chills<br>☐ Cough ☐ Shortness of breath<br>☐ Tired ☐ Wheezing<br>☐ Runny nose<br>☐ Other respiratory symptoms<br>☐ Joint pain ☐ Muscle pain<br>☐ Chest pain<br>☐ Headache ☐ Dizziness<br>☐ Nausea/vomiting<br>☐ Diarrhea ☐ Stomachache<br>Other: _________	☐ Yes ☐ No<br>☐ Unknown<br>*Specify month/year of illness:* ____/______<br>☐ Fever ☐ Chills<br>☐ Cough ☐ Shortness of breath<br>☐ Tired ☐ Wheezing<br>☐ Runny nose<br>☐ Other respiratory symptoms<br>☐ Joint pain ☐ Muscle pain<br>☐ Chest pain<br>☐ Headache ☐ Dizziness<br>☐ Nausea/vomiting<br>☐ Diarrhea ☐ Stomachache<br>Other: _________
Date of last symptom onset: (if participant is currently symptomatic)	(Day/month/year) _____/_____/________<br>☐ No symptoms	
Did the above symptoms require medical treatment?	☐ Yes ☐ No	
Did the above symptoms cause you to leave work/school?	☐ Yes ☐ No	
Did these symptoms require you to be hospitalized?	☐ Yes ☐ No	

5. *ECONOMIC DAMAGE OF COVID-19*

Choose the best option describes your job status? Choose ONE	☐ Working. Please specify occupation: ________________<br>☐ Self business. Please specify occupation: _____________<br>☐ Retired ► Move to **bold question**<br>☐ Housewife/childcare ► Move to **bold question**<br>☐ Pupils/Students ► Move to **bold question**<br>☐ Looking for a job ► Move to **bold question**<br>☐ Not working ► Move to **bold question**<br>☐ Other. Please specify: _________

What is your average monthly income?	_______________ Dong/month
If working, how many days have you been interrupted in the last 30 days?	Number of days interrupted: __________ day(s)
On average, how much money did you lose for not working each day?	_______________ Dong/day
**Total monthly income of your family members?**	
Before the epidemic:<br>☐ Less than 0.5 million Dong<br>☐ 0.5 – <1 million Dong<br>☐ 1.0 – <1.5 millions Dong<br>☐ 1.5 – <2.5 millions Dong<br>☐ 2.5 – <5.0 millions Dong<br>☐ 5.0 – <7.5 millions Dong<br>☐ 7.5 – <15 millions Dong<br>☐ 15 – <30 millions Dong<br>☐ >= 30 millions Dong<br>☐ No income<br>Specify: _______________ Dong	*In the last 30 days:*<br>☐ Less than 0.5 million Dong<br>☐ 0.5 – <1 million Dong<br>☐ 1.0 – <1.5 millions Dong<br>☐ 1.5 – <2.5 millions Dong<br>☐ 2.5 – <5.0 millions Dong<br>☐ 5.0 – <7.5 millions Dong<br>☐ 7.5 – <15 millions Dong<br>☐ 15 – <30 millions Dong<br>☐ >= 30 millions Dong<br>☐ No income<br>Specify: _______________ Dong
Is your family's current income enough to cover all expenses or do you receive additional money or support?	☐ Yes, our income is enough to spend<br>☐ No, we need to find additional sources.<br>(specify the offsets for this deficit)<br>_________________________
On average, how much money have you and your family lost over the last 30 days due to COVID-19 epidemic? (e.g. reduced wages, reduced number of customers, etc.)	Yourself: _______________ Dong<br>Family: _______________ Dong
Did you receive financial support for damages caused by the pandemic from any individual or organization?	☐ Yes ☐ No<br>If yes, specify name of individual, organization:<br>___________________________________<br>Amount of funding supported: _____________ Dong
On average, how much money have you spent on COVID-19 prevention? (eg: buy masks, buy sanitizers, etc.)	In the last 1 month: ____________________ Dong<br>In the last 3 months: ____________________ Dong<br>In the last 6 months: ____________________ Dong

5. COVID-19 ASSOCIATED KNOWLEDGE

Part 1: Please tick the answer box that you think is most correct about the COVID-19 disease information below.

**Statement**	**True**	**False**	**Unknown**
The main symptoms of COVID-19 are fever, dry cough, fatigue, and muscle pain.			
Currently, there is no specific treatment for COVID-19.			
People with chronic illnesses, the elderly, and people who are obese have greater risk of developing severe COVID-19 illness.			
Asymptomatic people infected with COVID-19 will not infect others.			
People in close contact or eating wild animals are likely to be infected with COVID-19.			
The virus that causes COVID-19 is transmitted mainly through droplets from an infected person.			
COVID-19 can be spread by touching eyes, nose, or mouth with hands contacted with infected surfaces (on which, the virus is attached)			
Children and young people do not need to practice measures against COVID-19 infection.			
Wearing mask when going to public places is very important in preventing COVID-19 infection.			
People should avoid going to crowded places (conferences, wedding parties, etc.) and avoid using public transport (buses, planes, etc.) to prevent COVID-19 infection.			
Washing hands with soap for at least 20 seconds can kill the virus.			
There is no need to clean surfaces regularly as the virus that causes COVID-19 does not survive long on surfaces.			
Isolating COVID-19 patients is an effective way to reduce the spread of the disease.			
People who come into contact with someone infected with COVID-19 should be isolated immediately. The quarantine period is 14 days.			

Part 2: Please tick the answer that best fit your opinion.

Question	Level			
	**High**	**Medium**	**Low**	**Very low**
What do you think your risk of contracting COVID-19 is?				
You think:	**Very easy**	**Easy**	**Difficult**	**Very difficult**
Washing your hands often with soap for at least 20 seconds is				
Washing your hands often with alcohol-based hand sanitizer is				
Avoiding touching eyes, nose and mouth with hands is				
Avoid going to crowded places is				
Covering your mouth when coughing or sneezing with elbow is				
Limiting contact with people who are sick is				
Standing >2m away from the person next to you is				
Wearing a mask whenever you go to public places is				
Self-isolation if you have any symptoms of illness is				

END OF INTERVIEW

References

1. Thomas SJ, Nisalak A, Anderson KB, Libraty DH, Kalayanarooj S, Vaughn DW, et al. Dengue plaque reduction neutralization test (PRNT) in primary and secondary dengue virus infections: How alterations in assay conditions impact performance. Am J Trop Med Hyg. 2009;81(5):825-33.
2. Nguyen CT, Moi ML, Le TQM, Nguyen TTT, Vu TBH, Nguyen HT, et al. Prevalence of Zika virus neutralizing antibodies in healthy adults in Vietnam during and after the Zika virus epidemic season: a longitudinal population-based survey. BMC Infect Dis. 2020;20(1):332.
3. Phan LT, Nguyen TV, Huynh LKT, Dao MH, Vo TAN, Vu NHP, et al. Clinical features, isolation, and complete genome sequence of severe acute respiratory syndrome coronavirus 2 from the first two patients in Vietnam. J Med Virol. 2020;92(10):2209-2215.
4. Vietnam General Statistics Office. Complete results of the 2019 Vietnam Population and Housing Census. Hanoi, Vietnam: Statistical Publishing House, 2010. Available at https://www.gso.gov.vn/du-lieu-va-so-lieu-thong-ke/2020/11/ket-qua-toan-bo-tong-dieu-tra-dan-so-va-nha-o-nam-2019/.
